# Bacteriological quality of raw camel milk along the market value chain in Fafen zone, Ethiopian Somali regional state

**DOI:** 10.1186/s13104-016-2088-1

**Published:** 2016-05-26

**Authors:** Tsegalem Abera, Yoseph Legesse, Behar Mummed, Befekadu Urga

**Affiliations:** Department of Veterinary Microbiology and Public Health, College of Veterinary Medicine, Jigjiga University, Jijiga, Ethiopia; Department of Clinical Studies, College of Veterinary Medicine, Jigjiga University, Jijiga, Ethiopia

**Keywords:** Raw camel milk, Microbiological quality, Milk value chain, Fafen zone

## Abstract

**Background:**

The camel is a multipurpose animal with a huge productive potential. Camel milk is a key food in arid and semi-arid areas of the African and Asian countries. The quality of milk is influenced by different bacteria present in milk. This study was conducted to evaluate total bacterial content in raw camel milk along the market chain in Fafen zone, Ethiopian Somali Regional State.

**Methods:**

One hundred twenty-six raw camel milk samples were collected from Gursum (47.1 %) and Babile (52.9 %) districts. The three sampling levels included were udder (14.7 %), milking bucket (29.4 %) and market (55.9 %). Milk samples were analyzed for total bacterial counts (TBC) and coliform counts (CC). Furthermore, major pathogens were isolated and identified.

**Result:**

108 (85.7 %) of raw camel milk samples demonstrated bacterial contamination. The overall mean TBC and CC of contaminated raw camel milk samples was 4.75 ± 0.17 and 4.03 ± 0.26 log CFU/ml, respectively. TBC increased from udder to market level and was higher in Gursum compared to Babile district (*P* < 0.05). Around 38.9 % of TBCs and 88.2 % CCs in contaminated raw camel milk samples were in the range considered unsafe for human utility. *Staphylococcus* spp. (89.8 %), *Streptococcus* spp. (53.7 %), *E. coli* (31.5 %), *Salmonella* spp. (17.6 %), *Klebsiella* spp. (5.6 %) and *Enterobacter* spp. (5.6 %) were the major bacterial microorganisms isolated.

**Conclusion:**

The majority of the bacterial isolates in this study showed high incidence in market as compared to production level. These results indicate a lack of compliance with good production practices and hygiene at milking, transportation and market of raw camel milk.

## Background

There are about 22 million camels in the world, of which 89 % are one-humped (*Camelus dromedarius*) camels and Ethiopia has 2.3 million camels [1] kept mainly by the Afar, Somali, Borana, Kerreyu, Beja and Rashaida pastoralists [2, 3]. With its unique bio-physiological characteristics, the dromedary has become an icon of adaptation to challenging ways of living in arid and semi-arid regions [4].

Camel milk is a key food in arid and semi-arid areas of the African and Asian countries. The milk is traditionally consumed predominantly in its raw or fermented form [5, 6]. In Ethiopia, most of the camel milk is consumed in the raw state without any heat treatments [7, 8]. Sour camel milk represents the major supply of food to settlements and towns in Ethiopian Somali Regional State [2]. Non-heat treated milk and raw-milk products as the major factors responsible for illnesses caused by food-borne pathogens as numerous epidemiological reports have implicated [9]. Contaminations can occur along the chain from producers to final consumers and the consumption of raw camel milk should be of major concern from public health point of view [10].

Milk is an excellent culture medium for the growth of microorganisms. The rate of multiplication of microbes depends mainly on storage temperature and time, level of nutrients and handling conditions. The external sources of microbes include the equipment, the personnel and water. The ability of microorganisms to cause spoilage and disease depends upon the type present, the initial load of contamination of the milk, handling conditions and the time lapse from production before consumption [11].

Raw camel milk may contain microorganisms pathogenic for man and the contamination can generally occur from three main sources; within the udder, outside the udder, and from the surface of equipment used for milk handling and storage. Pathogenic bacteria may present in raw milk as a direct consequence of udder disease. Total number of organism in milk as disease causative agent in relation to its proper evaluation for consumption is important. The notable disease causing bacteria in milk are *Salmonella*, *Brucella*, *Staphylococcus*, *Listeria* and coliforms. Coliforms are normal inhabitants of the large intestine and their presence in milk could indicate fecal contamination [12].

Quality of raw milk is a function of nutrition and health of the animal, chemical combination, and its microbial activities. The two dominant factors of the quality are the time before delivery to the consumer and condition of keeping the product. Microbial analysis of milk and milk products includes tests such as total bacterial count, yeasts and molds, and coliform estimation. High population of bacteria in aseptically drawn milk samples or detection of presence of harmful pathogenic microorganisms is an evidence of unhygienic milk production conditions [13, 14].

Camel milk production and consumption in Ethiopia was confined to the pastoral areas. In the recent past, it was introduced in the urban centers through informal marketing. Other communities have taken up the consumption of camel milk. There are no adequate hygienic practices in the camel milk production and processing since there are no quality standards set for camel milk in Ethiopia. This poses a high risk of microbial contamination and possible transmission of pathogenic microorganisms. The informal marketing of camel milk is a risk to consumers. Information on microbial quality and safety of camel milk procurement and marketing chain in peri-urban and urban markets is lacking and research outputs available on microbial evaluation of raw camel milk in Ethiopia is limited [14, 15], and to the best of our knowledge no work has been conducted at the various levels of the value chain in Somali regional state of Ethiopia. Therefore, the objectives of the present study were to assess the microbial quality of raw camel milk along the value chain in Fafen zone and to isolate and identify the major bacterial pathogens in the raw camel milk.

## Methods

### Study area

The current study was carried out in Babile and Gursum districts of Fafen Zone, Ethiopian Somali Regional State. Fafen zone is one of the nine administrative zones of the region. In Fafen Zone pastoralism, agro-pastoralism and sedentary production systems comprise 34.1, 56.8 and 9.1 %, respectively [16].

### Ethics

The work did not involve experimental animals or human subjects. As such it was exempted from institutional ethical clearance.

### Study design

A cross-sectional survey study design was employed to assess bacteriological quality and safety of camel milk at production and market level in Fafen zone.

### Sampling method

Dadhem, Dakata, Kubijara and Bombas areas were purposively selected from Gursum and Babile districts based on their high camel resources, camel milk marketing and accessibility. Camel herds which have lactating she-camel were selected in each area. Accordingly, 47 samples from udder, 22 from milking bucket and 57 from market were collected for the bacteriological milk quality studies.

**Fig. 1:**
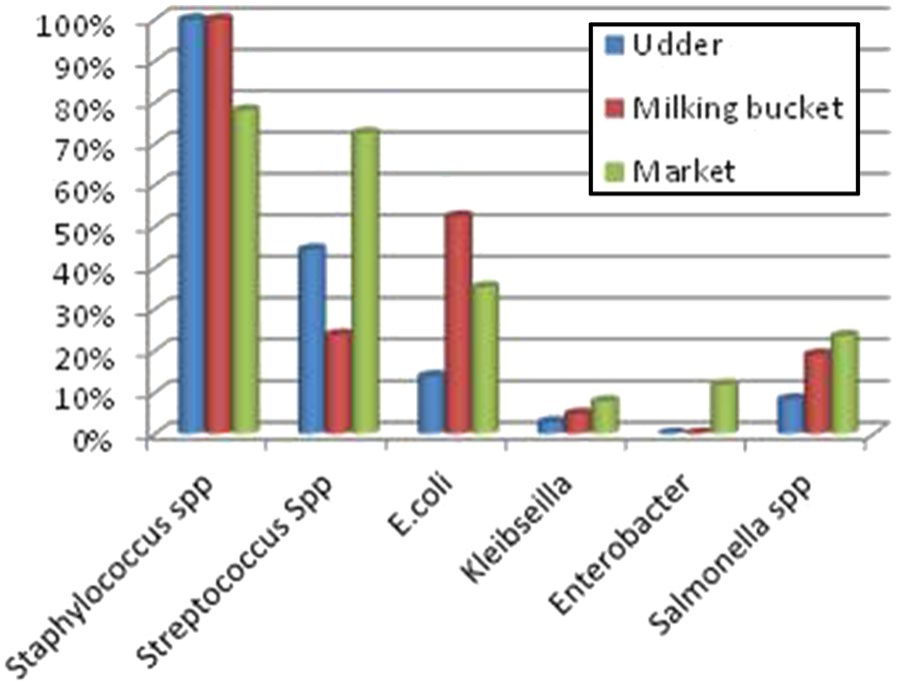
The total percentage of microorganisms associated with raw camel milk

### Milk sample collection

About 25 ml of fresh whole milk samples were collected from each three sampling points (directly from the udder of lactating camels, traditional milking buckets, market) by using sterile screw caped universal bottle. All samples were securely capped, labeled with permanent markers and kept below 10 ^°^C in a cool box that had cooling elements. The samples were transported to the laboratory and analysis started immediately. The microbiological analysis of the samples was done at the Microbiology Laboratory, College of Veterinary Medicine, Jigjiga University.

### Bacteriological analysis

The bacteriological tests considered for determination of the bacterial load in raw milk samples were total bacterial count (TBC) and coliform count (CC). For these two procedures standard plate count agar (Oxoid, UK) and violet red bile agar (HiMedia, India) were used, respectively. Peptone water was used for serial ten-fold dilutions.

### Standard plate count (SPC)

The TBC was done using pour plate method. Standard Plate Count Agar was used. This test was carried out to determine the content of microbial contamination of milk before any processing was done. One milliliter (1 ml) of milk sample was serially diluted in 9 ml of peptone water (ratio of 1:10) up to six dilutions. Sterile duplicate glass petri dishes were labeled according to the dilution index. One ml of the dilutions was aseptically withdrawn using a sterile 1 ml Pasteur pipette and delivered into an opened and sterile petri dish and then closed. The same was done for a duplicate petri dish. This was repeated till all the dilutions were pipetted into their corresponding plates up to 10^−6^. This was followed by pouring about 15 ml of standard plate count agar which had been autoclaved at 121 °C for 15 min, cooled and tempered in a water bath at 50 °C. The sample and the agar were gently mixed by alternate clock and anti-clockwise rotations and left to solidify on the bench for about 30 min. The plates were inverted and incubated at 37 °C for 48 h. After incubation, plates inoculated with sample dilution yielding between 30 and 300 colonies were counted. Colony counts were made using colony counter.

### Coliform count

One ml of milk sample was added into sterile test tube having 9 ml of peptone water. After mixing, the sample is serially diluted up to 1: 10^−5^ and 1 ml of inoculum was mixed thoroughly with molten 15–20 ml Violet Red Bile Agar (HiMedia, India) solution which was previously held in a water bath at 50 °C. Two plates were inoculated with each dilution. After thoroughly mixing, the plated sample is allowed to solidify and then incubated at 37 °C for 24 h. Typical dark red colonies are considered as coliform colonies. Finally, colony counts were made using colony counter.

In dishes which contain 30–300 colonies the actual number in both plates of a dilution was counted as per the formula given by [17].

### Bacterial isolation and identification

Characterization of bacterial isolates was carried out using colonial morphology, microscopic techniques and biochemical tests including gram’s reaction, coagulase test, oxides test, Oxidation–Fermentation test, catalase test and 3 % KOH tests. Highly selective media like Edwards Medium (HiMedia, India), Manitol Salt Agar(HiMedia, India), MacConkey agar (HiMedia, India), Eosin Methylene Blue agar (HiMedia, India), Xylose-lysine-deoxycholate medium (HiMedia, India), Brilliant Green Agar (HiMedia, India) and Salmonella Shigella Agar (HiMedia, India) were used. Triple Sugar Iron agar (HiMedia, India) was also used for differentiation of coliforms based on their ability of fermenting sugar and H_2_S production.

### Data management and analysis

Microsoft excel spread sheet was employed for raw data entry. Data on the bacterial counts was first transformed to logarithm of colony forming units per milliliter of sample (log CFU/ml) and the results were presented as mean ± standard error (SE) and percentage (%). Average TBC and CC content of positive samples was compared across districts (student t test) and sampling level (one way analysis of variance/ANOVA). Standard European Union (EU) microbiological limits (TBC ≤1 × 10^5^ CFU/ml and CC ≤10^2^ CFU/ml) for acceptable level of bacterial contamination in cow milk [18] were used to qualify contamination in raw camel milk samples. Variation in frequency of unacceptable TBC and CC between districts and sampling levels was evaluated using Chi square test. Chi square test was also used for testing variations in detection rate of specific milk contaminants across sampling level. Statistical significance was determined at *P* < 0.05.

## Results

A total of 126 raw camel milk samples were taken from three sampling levels namely directly from the udder, milking bucket and market from Gursum and Babile districts, out of which 108 (85.7 %) were found contaminated with aerobic bacteria including coliform bacteria 34 (27 %). The overall mean TBC and CC of raw camel milk samples was 4.75 ± 0.17 and 4.03 ± 0.26 log CFU/ml, respectively.

### Comparisons of the initial load of bacteria from different sources

Milk samples from Babile district had significantly higher mean TBC (*P* < 0.05) as compared to that of samples from Gursum district. Similarly, mean TBC showed a statistically significant (*P* < 0.05) increase from udder to market level (Table 1). No statistically significant variation was observed in CC in milk samples collected from the two districts. Meanwhile, CC demonstrated a limited increase (*P* > 0.05) from production to market level (Table 2).

**Table 1:** Mean ± Standard error values of total viable counts from different sources

Parameter		Mean ± SE TBC (log CFU/ml)	P value
District	Babile	4.35 ± 0.19	0.005
	Gursum	5.30 ± 0.29	
Sampling level	Udder	4.20 ± 0.3	0.039
	Milking bucket	4.8 ± 0.4	
	Market	5.1 ± 0.2

**Table 2:** Mean ± Standard error values of coliform counts from different sources

Parameter		Mean ± SE CC (log CFU/ml)	P value
District	Babile	3.8 ± 0.2	0.311
	Gursum	4.3 ± 0.5	
Sampling level	Udder	3.5 ± 0.4	0.455
	Milking bucket	3.7 ± 0.5	
	Market	4.3 ± 0.4	

Out of 108 samples positive for aerobic bacteria, 38.9 % had TBCs of greater than the minimum acceptable level for cow milk whereas 61.1 % raw milk samples had TBCs within the acceptable threshold [18]. The frequency of raw milk TBC in the unacceptable range increased (*P* < 0.05) from production to market level (Table 3). Out of 34 coliform positive raw camel milk samples 30 (88.2 %) had unacceptable contamination levels [18]. Unacceptable level of coliform contamination increased (*P* < 0.05) from production to market level (Table 4).

**Table 3:** Total aerobic bacterial count in different sampling points

Sampling point	No of samples	Total aerobic bacterial count (CFU/ml)	
		≤10^5^	>10^5^
Udder	36	27 (75.0 %)	9 (25.0 %)
Milking bucket	21	15 (71.4 %)	6 (28.6 %)
Market	51	24 (47.1 %)	27 (52.9 %)

χ^2^ = 10.9; P value = 0.004

**Table 4:** Total coliform count in different sampling points

Sampling point	No of samples	Total coliform count (CFU/ml)	
		≤10^2^	>10^2^
Udder	5	0	5 (100 %)
Milking bucket	10	4 (40 %)	6 (60 %)
Market	19	0	19 (100 %)

χ^2^ = 7.9, P value = 0.019

### Types of microorganisms in raw camel milk

The type of bacteria isolated from contaminated raw camel milk samples include; *Staphylococcus* spp. (89.8 %), *Streptococcus* spp. (53.7 %), *E. coli* (31.5 %), *Klebsiella* spp. (5.6 %), *Enterobacter* spp. (5.6 %) and *Salmonella* spp. (17.6 %) (Fig. 1). *Staphylococcus* spp. showed the highest prevalence at production level whereas that of *Streptococcus* spp. and coliforms tend to increase from production to market level (Table 5).

**Table 5:** The frequency distribution of the organisms in raw camel milk

	Sampling levels			P value
	Udder	Milking bucket	Market	
*Staphylococcus* spp.	36 (100 %)	21 (100 %)	40 (78 %)	0.001
*Streptococcus* spp.	16 (44.4 %)	5 (23.8 %)	37 (72.5 %)	0.000
*E. coli*	5 (13.9 %)	11 (52.4 %)	18 (35.3 %)	0.007
*Klebsiella* spp.	1 (2.8 %)	1 (4.8 %)	4 (7.8 %)	0.750
*Enterobacter* spp.	0	0	6 (11.8 %)	0.039
*Salmonella* spp.	3 (8.3 %)	4 (19 %)	12 (23.5 %)	0.189

## Discussion

Majority of raw camel samples were contaminated by different bacteria with samples demonstrating marked variability in level of contamination. It is worth to mentioning that there are currently no microbiological standards concerning camel milk. Therefore, standard European Union (EU) microbiological limits (TBC ≤ 1 × 10^5^ CFU/ml and CC ≤ 10^2^ CFU/ml) for acceptable cow milk [18] were used to assess the quality of camel milk in this study.

TBC is a good indicator for monitoring the sanitary conditions practiced during production and handling of raw milk. The mean raw camel milk TBC observed in this study agrees with those reported by [19] (5.0 log CFU/ml), [15] (5.6–5.0 log CFU/ml), [20] (5.4 log CFU/ml), [21] (5.22 log CFU/ml) and [22] (3.0–5.0 log CFU/ml). The current mean TBC was in the range of EU acceptable limits for raw milk intended for direct human consumption and processing. However, milk samples collected from Gursum district showed slightly above the recommended limit [18]. This might be due to the differences in initial contamination originating from the udder surface, quality of water used for cleaning milking utensils and the time lapse from production to marketing. Milk collected directly from udder and milking bucket was found with relatively better bacteriological quality than the milk collected from market. This might be due to the traditional methods of distribution and transportation of milk including; use of easily contaminated and hard to clean container, long transit time to markets with frequent opening of containers for retail or milk transfer.

The mean CC observed in the current study is higher than the value of 2.83 log CFU/ml reported for milk samples collected from camels in central and southern regions of United Arab Emirates [22]. However, it was lower than that reported by [23] 6.85 log coliform CFU/ml in Morocco and [24] in south west Algeria (6.75 log coliform CFU/ml). The overall value of coliform counts observed in the current study was much higher when compared with the recommended values given by the American Public Health Association and EU (<100 CFU/ml). Mean CC increased in camel milk shows relative increase from udder to milking bucket to market. This might be due to milk contamination at different levels while milk was passing through different stages of production. The presence of high numbers of coliforms in milk indicates that the milk has been contaminated with fecal materials and it is an index of hygienic standard used in the production of milk. This could be attributed to insufficient pre-milking udder preparation, poor hand washing practice of milker and use of poor quality and non-boiled water for cleaning of milking utensil. Coliforms, when present in any food, signal possibility of enteric pathogens and unhygienic conditions under which the food was produced and handled. The increase in coliforms in the market raw camel milk could be associated with contaminated containers, water and the soil. Transferring of milk from container to the next during bulking towards the market makes milk sweep over wide container surfaces, thus collecting the microorganisms from container surfaces [25].

The present study revealed that *Staphylococcus* spp. and *Streptococcus* spp. are the dominant bacteria isolated in raw camel milk samples. The result is in agreement with [19] who reported that nearly 70 % (n = 23) of camel milk samples are contaminated with *Staphylococcus aureus*. This could be due to poor hygienic practices and presence of subclinical mastitis. 31.5 % of the raw camel milk samples under study were contaminated with *E. coli*. This agrees with [26] who reported 39.13 % in camel milk collected from Bahrei area in the Sudan. The reason could be due to contamination of the milk samples from the camel, the milkers, milk containers and the milking environment. The incidence of *Salmonella* spp. in this study was high. The result was in agreement with that of 24 % reported [19]. 13 % was reported for *Salmonella enterica* occurrence along the camel milk chain in Kenya [26]. These organisms pose a health risk to consumer if milk is consumed without any heat treatment. The majority of the bacterial isolates in this study showed high incidence in market as compared to production level. The increase in frequency of the isolates at market centers can be associated with post-harvest handling of the milk.

## Conclusion

Results from the present study clearly indicated that the microbial quality and safety of raw camel milk at various levels of value chain in Babile and Gursum districts is low. Significant differences were observed in bacteriological quality in camel milk samples along the value chains in which high degree of contamination occurred at sale points than at farm level. The total coliform count obtained in the present study was higher than acceptable limits. The presence of these coliform bacteria not only indicates the poor hygienic conditions in which milk is produced and marketed but also they could be pathogenic. The major isolates were *Staphylococcus* spp., *Streptococcus* spp., *E. coli* and *Salmonella* spp. Therefore, strict hygienic control measures along the value chain to improve hygienic conditions of milk from production to consumption should be implemented and the work on the determination of camel milk standards in Ethiopia should be initiated.

## Abbreviations

TBC: total bacterial count
CC: coliform count
SPC: standard plate count
CFU: colony forming unit
EU: European union
